# Adenosine Triphosphate in Serum as a Promising Biomarker for Differential Diagnosis of Hepatitis B Disease Progression

**DOI:** 10.3389/fimmu.2022.927761

**Published:** 2022-07-01

**Authors:** Caorui Lin, Ying Huang, Linjie Luo, Fengling Fang, Jiawei Zhang, Zhen Xun, Ya Fu, Hongyan Shang, Can Liu, Qishui Ou

**Affiliations:** ^1^ Department of Laboratory Medicine, Gene Diagnosis Research Center, The First Affiliated Hospital, Fujian Medical University, Fuzhou, China; ^2^ Fujian Key Laboratory of Laboratory Medicine, The First Affiliated Hospital, Fujian Medical University, Fuzhou, China; ^3^ First Clinical College, Fujian Medical University, Fuzhou, China

**Keywords:** adenosine triphosphate (ATP), hepatitis B virus (HBV), chronic hepatitis B (CHB), mitochondria, HBV infection, progression

## Abstract

The need to be diagnosed with liver biopsy makes the clinical progression of chronic HBV infection diagnosis a challenge. Existing HBV serum biochemical assays are used throughout clinical but have limited effects. Studies have shown that mitochondrial function is tightly coupled to HBV infection. Here, we verified the diagnostic value of serum Adenosine Triphosphate (ATP) as a potential marker for differential HBV infection progress by detecting the level of ATP in the serum from a wide spectrum of HBV-infected populations, and confirmed the role of ATP in the deterioration of HBV infection-related diseases through HBV-infected cells and mouse models. The results showed that there were significantly lower serum ATP levels in HBeAg-positive CHB patients compared with healthy controls. And during the progression of CHB to liver cirrhosis and hepatocellular carcinoma, the ATP level was increased but not higher than healthy controls. The area under the curve (AUC) of serum ATP was 0.9063 to distinguish HBeAg-positive CHB from healthy, and another AUC was 0.8328 in the CHB against the HCC group. Preliminary exploration of the mechanism indicated that the decline of serum ATP was due to impaired mitochondria in CHB patients. Our data provide evidence that serum ATP distinguishes the various progress of HBV infection-related diseases and expands diagnostic biomarkers for HBeAg-positive CHB patients with healthy controls.

## Introduction

Chronic hepatitis B (CHB) remains a global public health concern that potentially life-threatening liver infection caused by the hepatitis B virus (HBV). There are about 257 million chronic hepatitis B virus (HBV) infected people in the world, and approximately 887000 deaths every year from HBV infection-related diseases according to the report by the World Health Organization (1). It is currently a moderate endemic area of ​​HBV infection in China, and the rate of positive HBsAg is 5% to 6% in the general population. There are about 70 million cases of chronic HBV infection, including about 20 million to 30 million cases of chronic hepatitis B (2).

Chronic Hepatitis B (CHB) represents a long-term chronic invasion of HBV that the course of disease is more than half a year or the date of onset is unclear, manifest in the clinical characteristic of abdominal distension, fatigue, loss of appetite, liver pain and other symptoms (3). CHB can progress to cirrhosis of liver (LC) and even hepatocellular carcinoma (HCC) without prompt and effective treatment, and existing antiviral treatment methods cannot prevent the occurrence of LC and HCC (4). Therefore, it is of great importance for the disease assessment and medication guidance of CHB patients to regularly monitor the progress of disease. At present, HBV serum markers including HBsAg, anti-HBs, HBeAg, anti-HBe, anti-HBc, and anti-HBc IgM are mainly used to measure HBV replication ability in clinical and combine with biochemical indicators (including serum albumin, alanine aminotransferase (ALT) and aspartate aminotransferase (AST), etc.) and imaging diagnosis, and other emerging methods such as FibroBox (5) and identification of serum lipid alterations (6) to evaluate liver function and liver damage. However, the above detection indexes have remained insufficient in the determination with great precision of the progress of CHB (7). It is urgent for the diagnosis and progress assessment of CHB patients to investigate new markers or develop other combined diagnostic methods.

The liver injury of CHB patients is the pivotal result from infiltration of inflammatory cells and metabolic disturbances of hepatocytes due to HBV infection (4, 8). The liver is the central organ of metabolism that contains more abundant mitochondria than other tissues, the number of which is 500–4000 mitochondrion per cell, occupying about 20% of the entire liver cell volume (9). Energy generation and the regulation of cell metabolism are the most important functions of mitochondria, which also can control antiviral immunity, oxidative stress, cell cycle regulation, and apoptosis (10). Previous studies have demonstrated that HBV infection can induce mitochondrial dysfunction, including the depletion of mtDNA, impaired activity of respiratory chain complexes, and abnormal morphological alteration, which in turn results in liver damage and accelerating CHB progression (11, 12).

Recent studies have shown that HBV infection can disrupt the function of mitochondrial complexes I, III, IV, and V (13, 14), leading to the dysfunction of proton transport, accumulation of excess protons and electrons, and subsequent reduction of Adenosine Triphosphate (ATP) production (15, 16). However, the alteration of serum ATP in the progression of CHB has not been reported. In this study, we evaluated the correlation between serum ATP and HBV infection by measuring the levels of serum ATP in patients at different stages of HBV infection. Then we verified the association in HBV infected cells and mice and proved that mitochondrial damage elicited by HBV infection is the major reason for serum ATP decline. Finally, we used the ROC curve to analyze the diagnostic value of serum ATP in CHB patients. This article aimed to evaluate the correlation between serum ATP with the progress of HBV infection, to provide new diagnostic markers or predictors for the degree of disease progression in CHB patients.

## Results

### Serum ATP Is Significantly Decreased in Patients With HBeAg-Positive CHB

**Figure 1:**
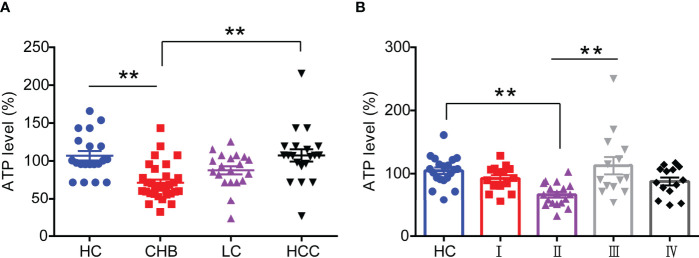
Serum ATP is significantly decreased in patients with HBeAg-positive CHB. **(A)** The level of serum ATP from healthy control (HC) (n=20), chronic hepatitis B (CHB) (n=32), liver cirrhosis (LC) (n=20), and hepatocellular carcinoma (HCC) (n=20). **(B)** The level of serum ATP across the natural history of chronic HBV infection, HC (n=20), HBeAg-positive chronic HBV infection (I) (n=15), HBeAg-positive CHB (II) (n=20), HBeAg-negative chronic HBV infection (III) (n=15), and HBeAg-negative CHB (IV) (n=15). Data are presented as means ± SEM, **p < 0.001; one-way ANOVA *post-hoc* Student–Newman–Keuls test.

substantial studies have shown that mitochondrial function is impaired in HBV infection-related diseases (11), but the expression levels of its associated derivatives such as ATP in HBV-infected patients have remained unknown. In this study, we enrolled and collected sera from 20 healthy controls (HC), 32 chronic hepatitis B (CHB) patients, 20 liver cirrhosis (LC) patients, and 20 hepatocellular carcinomas (HCC) patients and the details were depicted in **Table 1 in Supplementary Materials**, respectively. Then we analyzed the ATP expression levels in serum. As shown in **Figure 1A**, the ATP levels in the serum from CHB patients were significantly decreased compared with healthy controls. It was surprising that the disease progressed to LC and HCC induced a modest increase in serum ATP levels, especially in patients with HCC, but not distinguish from healthy controls. To determine the expression of serum ATP levels from patients in the process of HBV infection, we performed an investigation on patients from four different disease stages in HBV infection (**Table S2**). The results showed that compared with healthy controls, the serum ATP levels of CHB patients with HBeAg-positive (II) were obviously decreased, however, were not significantly different from HBeAg-positive chronic HBV infection (I) and HBeAg-negative CHB (IV). In addition, serum ATP dramatically rose in the progression from HBeAg-positive CHB (II) to HBeAg-negative chronic HBV infection (III) (**Figure 1B**). These data demonstrated that there was a variation in serum ATP levels in different stages of HBV infection and related liver disease progression.

**Figure 2:**
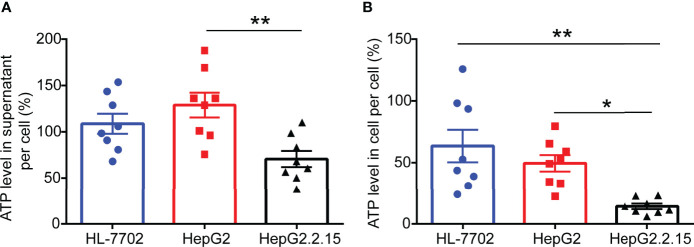
ATP levels are remarkably reduced in HepG2.2.15 cell. **(A)** The level of ATP in the supernatant of per cell from HL-7702 cell, HepG2 cell, and HepG2.2.15 cell (n=8). **(B)** The level of ATP in per HL-7702 cell, HepG2 cell, and HepG2.2.15 cell (n=8). Data are presented as means ± SEM, *p < 0.05, **p < 0.001; one-way ANOVA *post-hoc* Student–Newman–Keuls test.

### ATP Levels Are Remarkably Reduced in HepG2.2.15 Cells

To investigate the effect of HBV infection on ATP expression levels, we selected a normal human liver cell line (HL-7702), human hepatoblastoma cell line (HepG2), and HepG2-derived cell line (HepG2.2.15) (17) that has stable HBV expression and replication to carry out ATP analysis. After 2 days of cell culture, we gathered cell supernatant and cell contents for ATP detection. As expected, higher levels of ATP in the supernatant of HepG2 cells were observed compared with HepG2.2.15 cells. Correspondingly, comparable levels of ATP were consistently found in the intracellular. The above results indicated that HBV invasion can elicit ATP decline in cells.

### HBV Elicits ATP Decline in HBV-Infected Mice

**Figure 3:**
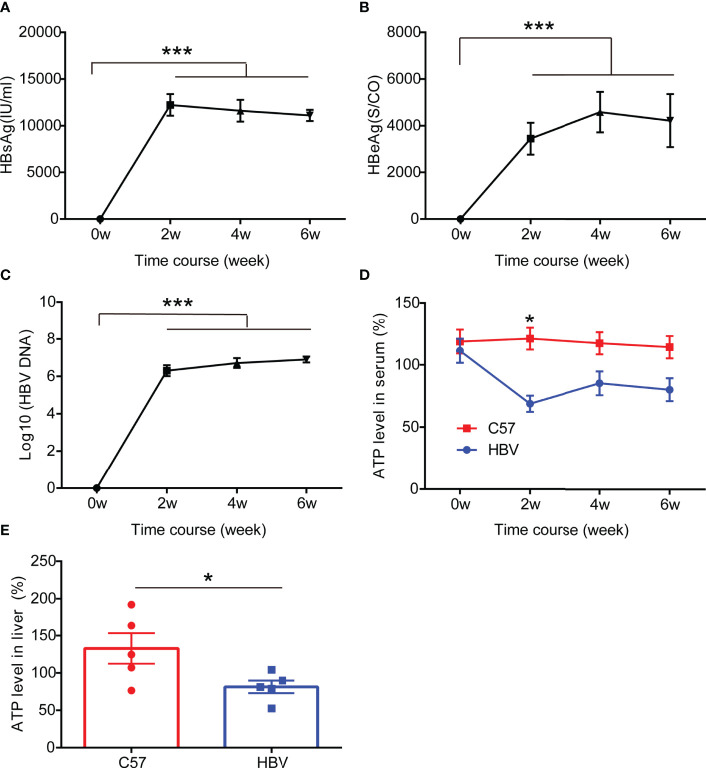
HBV elicits ATP decline in HBV-infected mice. **(A–C)** The representation of HBV markers in HBV-infected C57BL/6 mice including HBsAg, HBeAg, and HBV DNA (n=5). **(D)** The level of ATP in serum from HBV-infected C57BL/6 mice and age and gender matched non-HBV-infected C57BL/6 mice (n=5). **(E)** The level of ATP in the liver from HBV-infected C57BL/6 mice and age and gender matched non-HBV-infected C57BL/6 mice (n=5). Data are presented as means ± SEM, *p < 0.05, ***p < 0.0001; one-way ANOVA *post-hoc* Student–Newman–Keuls test.

To further evaluate the effect of HBV infection on the expression of ATP *in vivo*, we constructed a mouse model of HBV infection (18, 19), and serum was collected every two weeks to detect HBV replication and ATP levels. Unsurprisingly, consistent with previous observations on patients and cells, a significant drop of ATP levels in serum was detected in the HBV replication mouse model which could stably express HBV replication-related markers including HBsAg, HBeAg, and HBV DNA, particularly in the second week (**Figure 3**). At the end of the sixth week, we harvested liver tissue for ATP. And the results showed that the levels of ATP in the liver decreased significantly, which was half the level of wild-type mice (**Figure 3E**).

### HBV Lower ATP Levels by Disrupting the Development of Mitochondria

**Figure 4:**
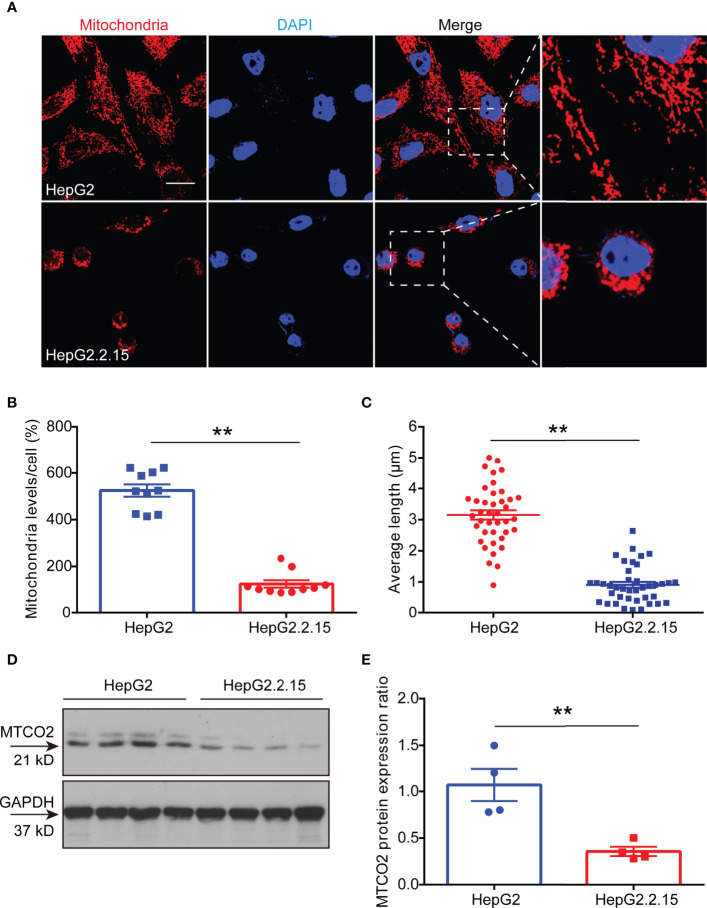
HBV lower ATP levels by disrupting the development of mitochondria. **(A)** Immunocytochemistry for mitochondria in HepG2 cells and HepG2.2.15 cells (scale bar, 10μm). **(B)** Quantitative analysis of mitochondrial level per cell in HepG2 cells and HepG2.2.15 cells (n=10). **(C)** Quantitative analysis of average mitochondrial branch length (μm) (n=40). **(D, E)** Western blot to detect **(D)** and quantitative analysis **(E)** of MTCO2 expression in HepG2 cells and HepG2.2.15 cells (n=4). Data are presented as means ± SEM, **p < 0.001; one-way ANOVA *post-hoc* Student–Newman–Keuls test.

Studies have shown that HBV infection compromises mitochondrial function (13, 20). To verify whether the decrease in ATP induced by HBV infection was due to mitochondrial destruction, we labeled mitochondria in HepG2 and HepG2.2.15 cells with Mito Tracker dyes. The results showed that the mitochondria in HepG2 cells were faultless, which represented in the shape of branched, circular, linear, or flat disc, and were abundant, while the mitochondria in HepG2.2.15 cells were characterized as broken, fragmented, and aggregated into spheres (**Figure 4A**), manifested in a reduction in its number (**Figure 4B**) and length (**Figure 4C**). Importantly, we found that the expression of cytochrome c oxidase subunit 2 (MTCO2) in HepG2.2.15 cell, a hallmark protein in mitochondria, which is a component of the cytochrome c oxidase, the last enzyme in the mitochondrial electron transport chain that drives oxidative phosphorylation and mediates ATP production, was also significantly reduced compared with which in HepG2 cell (**Figures 4D, E**). These studies have proved that the decrease of ATP in the serum is the result of mitochondrial destruction induced by HBV infection.

### Serum ATP Contributes to Diagnosing CHB and Predicting Its Poor Clinical Outcome

**Figure 5:**
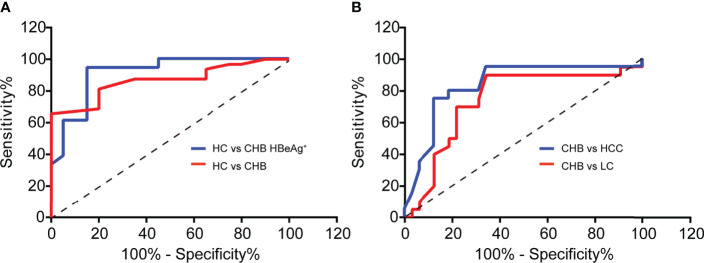
Serum ATP contributes to diagnosing CHB and predicting its poor clinical outcome. **(A)** ROC curves to validate the diagnostic value of Healthy control (HC) (n = 20) against chronic hepatitis B (CHB) (n = 32) (*p* < 0.0001) or HBeAg-positive CHB (n = 20) (*p* < 0.0001). **(B)** ROC curves to validate the diagnostic value of CHB (n=32) against liver cirrhosis (LC) (n = 20) (*p* = 0.0039) or hepatocellular carcinoma (HCC) (n = 20) (*p* < 0.0001).

Then we used the ROC curve to analyze the diagnostic value of serum ATP level in CHB patients against healthy control (HC). As shown in **Figure 5A**, the area under the curve (AUC) in the CHB against the HC group was 0.8656 with a 95% confidence interval (CI) ranging from 0.7686 to 0.9627. Notably, the AUC between HC and CHB of positive HBeAg group was 0.9063 (95% CI: 0.8104 to 1.002). Moreover, CHB will progress to LC or even HCC due to the lack of complete cure for CHB, and it can only be precisely diagnosed with liver biopsy. Therefore, we tried to perform the ATP level in serum as a predictor of the clinical outcomes of CHB, and we used the ROC curve to evaluate its predictive value. As expected, we found that ATP in serum can significantly distinguish CHB from LC or HCC. And we can detect the AUC in the CHB against the LC group was 0.7398 (95% CI: 0.5923 to 0.8874), and another AUC was 0.8328 (95% CI: 0.7099 to 0.9558) in the CHB against HCC group (**Figure 5B**). These results unveiled that serum ATP represented a robust association with the hepatitis B disease progression.

## Materials and Methods

### Patients and Healthy Participants

A total of 177 participants were enrolled from the First Affiliated Hospital of Fujian Medical University in the study, including 40 healthy controls (HCs) and 137 patients with HBV infection (**Figure S1**). The patients were classified into four phases based on the natural history of the chronic HBV infection (21), which respectively were HBeAg-positive chronic HBV infection, HBeAg-positive chronic hepatitis, HBeAg-negative chronic HBV infection, and HBeAg-negative chronic hepatitis. The characteristics of patients are summarized in **Tables S1**, **S2**.

The study was carried out in compliance with the 1975 Declaration of Helsinki and was approved by the Ethics Committee of the First Affiliated Hospital of Fujian Medical University (Approval No. MRCTA, ECFAH of FMU [2022]083). Written informed consent was obtained from the study participants.

### ATP Assay

The detection of ATP level in serum and cell were applied according to The CellTiter-Glo^®^ 2.0 Assay kit or RealTime-Glo™ Extracellular ATP Assay kit (Promega, WI, USA). The extraction of ATP from livers was the same as described previously (22). Briefly, snap-frozen livers were cut out into slices, and 10–20 mg of 4- to 6-μm-thick cryosections were collected into a 1.5-mL Eppendorf tube. Then 0.4 M HClO_4_ (600 μL) was added to dissolve sections with vortexing for 1 min on ice. The tube was spun for 5 min at 2000 rpm at 4°C, next the supernatant was transferred to a new tube and repeated the previous step to remove debris, then it was prepared to measure the ATP by CellTiter-Glo Luminescent Cell Viability Assay kit (Promega, WI, USA).

### Clinical Biochemistry

HBV DNA was detected using quantitative real-time PCR (Sansure Biotech Inc., Hunan, China) and a Roche Light cycler 480 (Roche Corporation, Basel, Switzerland). HBsAg and HBeAg were quantified using an automated chemiluminescent microparticle immunology analyzer (Abbott I2000, Abbott Laboratories, Chicago, IL, USA). Alanine aminotransferase (ALT) was quantified using an automatic biochemical analyzer ADVIA 2400 (Siemens, Munich, Germany).

### Cell Culture

HepG2 cells or HepG2.2.15 cells were kept in-house and cultured at 37°C in 5%CO_2_ in Dulbecco’s modified Eagle’s medium (DMEM) supplemented with 10% fetal bovine serum (FBS) and 1% penicillin and streptomycin. HepG2 cells (5× 10^3^) or HepG2.2.15 cells (5× 10^3^) were seeded in 96-well plates for 48 h prior to ATP assay.

### Establishment of a Recombinant Adeno-Associated Virus Type 8 (rAAV8)-Mediated HBV Replication Mouse Model

The 6-8week C57BL/6 mice were used in all experiments (the number of mice is specified in corresponding figure legends). Mice were housed under specific pathogen-free conditions in a temperature-controlled room. The experiments were performed according to the committee guidelines and approved by the Animal Experiment Center of the Fujian Medical University (IACUC FJMU 2022-0171). rAAV8 carrying the 1.3-mer wild-type HBV genome (rAAV8-1.3HBV) was purchased from the FivePlus Molecular Medicine Institute (Beijing, China) and was used to construct an immunocompetent mouse model for chronic HBV infection (19). Briefly, A total of 5×10^10^ viral genomes/200 μl virus were injected into the tail vein of the C57BL/6 mouse. The mice were bled every other week for 6 weeks to monitor the HBsAg, HBeAg, and HBV DNA levels. Mice were killed by CO2 inhalation at the sixth week, and liver tissues were snap-frozen in liquid nitrogen-cooled isopentane and stored at -80°C.

### MitoTracker Staining for Mitochondria

HepG2.2.15 cells (1×10^4^) or HepG2 cells (1×10^4^) were seeded on Matrigel-coated glass coverslips in 24-well plates for 24 h prior to the replacement with growth medium containing 300 nM MitoTracker dye (Thermo Fisher Scientific, USA) for 45 mins. Cells were fixed with 4% PFA for 30min at RT and permeabilized with 0.5% Triton X-100 in DPBS for 10 min, followed by rinsing with DPBS three times and blocking with 20% goat serum in DPBS for 90 min. Coverslips were mounted in fluorescent mounting medium (Dako) with 0.1% DAPI and visualized with confocal fluorescence microscopy (LSM510, Zeiss, Germany). ImageJ and Image-Pro Plus software (USA) were used for quantification analyses.

### Protein Extraction and Western Blot

For the detection of MTCO2 in HepG2 and HepG2.2.15 cells, total protein was extracted from the cell in culture (6×10^7^) by RIPA lysis buffer with 1mM PMSF. And the protein concentration was quantified by a Bradford assay (Sigma, USA). 50 μg protein were loaded onto SDS-polyacrylamide electrophoresis gels (4% stacking and 10% resolving), followed by transfer to the polyvinylidene fluoride (PVDF) membrane. The membrane was then washed and blocked with 5% skimmed milk and probed overnight with primary antibodies, including MTOC2 (1:5000, Abcam, UK) and GAPDH (1:1000, Cell Signaling Technology, USA). Secondary horseradish peroxidase (HRP)-tagged antibody (1:2000, Cell Signaling Technology, USA) was used and signals were visualized with an enhanced chemiluminescence (ECL) western blotting analysis system (Amersham Pharmacia Biosciences). Bands were quantified by densitometric analysis using the ImageJ software program (NIH, USA).

### Data Analysis

All data are expressed as mean values ± SEM unless indicated otherwise. Statistical differences between different groups were evaluated by SigmaStat (Systat Software, Chicago, IL, USA). Both parametric and non-parametric analyses were applied as specified in figure legends. Significance was determined based on *p*< 0.05.

## Discussion

HBV infection is known to induce dysfunction of mitochondria. In this study, we prove that the serum ATP associates with the progression of CHB. Significant decline levels of ATP were achieved in HBV infected cells and mice. Dissection of this correlation revealed that HBV infection destroys mitochondrial function, which in turn results in decreased ATP generation. Importantly, we demonstrate that serum ATP has a profound diagnostic value in HBeAg-positive CHB patients against healthy controls, and significantly distinguishes CHB from the other two outcomes of CHB. Our study provides a new diagnostic marker for the CHB progress and also insight into the role of ATP.

Mitochondrial function is severely impaired by HBV infection (10). In our previous study, we have shown higher levels of mtDNA in the serum of CHB patients than in healthy control. Currently, we further evaluated the ATP level during the progression of CHB, the results showed that serum ATP level was significantly decreased in HBeAg-positive CHB patients compared with healthy controls. Interestingly, we also found that serum ATP level of LC and HCC patients were decreased compared with healthy controls, but were slightly higher than the CHB patients. We supposed that the infiltration of inflammatory cells leads to massive hepatocyte necrosis and metabolic disorder at the HBeAg-positive CHB stage, which in turn compromises for mitochondrial function and ATP production. However, once processing into the stage of LC and HCC, collagen fibers and cancer cells begin to proliferate actively, which results in increasing ATP production in a feedback manner, and this is also consistent with the related energy consumption research of cancer cell (23). Further studies with expanded serum samples would be warranted to determine whether hepatocellular carcinoma can increase serum ATP levels.

As expectedly, we also detected the same decreased ATP level in HBV infected cells and mice, including in the serum and liver of mice. The association between ATP level and HBV infection was been further confirmed by the HBV infection model. Although we have not established a cell model of HBV transient infecting, the mouse model of HBV acute infection has been able to simulate the CHB stage well (17).

Another important finding from our current study is that we demonstrated that the declining level of serum ATP is the result from impaired mitochondrial shape and faulty oxidation respiratory chain. As reported in the previous studies, HBV infection disrupts mitochondrial function and affects cell metabolism, thereby hindering the production of ATP. In the subsequent work, if the liver tissues from CHB patients can be collected, we will try to examine the mitochondrial function in the liver tissue in order to confirm the reasons for the decline of serum ATP levels in CHB patients. Serum ATP is a comprehensive result from systemic metabolism, we think that the decrease in ATP in serum is a direct result of CHB in our article. One of the reasons, as you noted, we have demonstrated that ATP levels in HBV-infected cells (**Figure 2**) and liver tissues (**Figure 3**) are decreased *in vitro* and *in vivo* experiments. In addition, we also showed that HBV infection can disrupt mitochondrial function (**Figure 4**), which is the main source of ATP production. However, does the long-term presence of CHB lead to other feedbacks in the body, such as muscle dysfunction, which is another major source of ATP? In the future experiments, we may be able to examine ATP levels in muscle tissue after HBV infection. However, currently in this investigation, we believe that the ATP decline is mainly driven by CHB.

One major hurdle of the clinical diagnostic for CHB patients is the need to be cooperated with liver biopsy (5), which is not accepted by most patients. In this research, we have proved the diagnostic value of serum ATP in the CHB patients by ROC curve analysis. We have confirmed that the level of serum ATP can be used to diagnose the HBeAg-positive CHB against healthy control, and predict the poor clinical outcome of CHB patients. In the detailed further studies, we can prepare some ATP standards to perform absolute quantification of ATP, and combine them with the existing CHB detection indicators to comprehensively evaluate the diagnostic value of ATP in CHB patients compared with liver biopsy.

In summary, our results demonstrated that serum ATP robustly associates with the progression of CHB, and would be a promising biomarker for the diagnosis of HBeAg-positive CHB patients and healthy control.

## Data Availability Statement

The original contributions presented in the study are included in the article/**Supplementary Material**. Further inquiries can be directed to the corresponding author.

## Ethics Statement

The studies involving human participants were reviewed and approved by the Ethics Committee of the First Affiliated Hospital of Fujian Medical University (Approval No. MRCTA, ECFAH of FMU [2022]083). The patients/participants provided their written informed consent to participate in this study. The animal study was reviewed and approved by Animal Experiment Center of the Fujian Medical University (IACUC FJMU 2022-0171).

## Author Contributions

CRL, and QSO conceived the study questions and designed the study. CRL, YH, LJL, FLF, and JWZ performed the experiments and analyses. ZX, and YF collected samples and laboratory data. HYS, CL,and QSO provided materials. CRL drafted the manuscript. QSO supervised the study. All authors contributed to the article and approved the submitted version.

## Funding

This work was supported by the National Natural Science Foundation of China (Grant number 82030063), the Joint Funds for the Innovation of Science and Technology, Fujian Province (Grant number 2019Y9017), and the Natural Science Foundation of Fujian Province (Grant number 2022J05051).

## Conflict of Interest

The authors declare that the research was conducted in the absence of any commercial or financial relationships that could be construed as a potential conflict of interest.

## Publisher’s Note

All claims expressed in this article are solely those of the authors and do not necessarily represent those of their affiliated organizations, or those of the publisher, the editors and the reviewers. Any product that may be evaluated in this article, or claim that may be made by its manufacturer, is not guaranteed or endorsed by the publisher.
