# Supplementary material for: Adenosine Triphosphate in Serum as a Promising Biomarker for Differential Diagnosis of Hepatitis B Disease Progression
Source: Front Immunol. 2022 Jul 1;13:927761. doi: 10.3389/fimmu.2022.927761 (PMC9284211; doi:10.3389/fimmu.2022.927761)
Supplement: Supplementary file 1 [file DataSheet_1.doc]

**FIRURE S1 Flowchart of the patient selection process.**


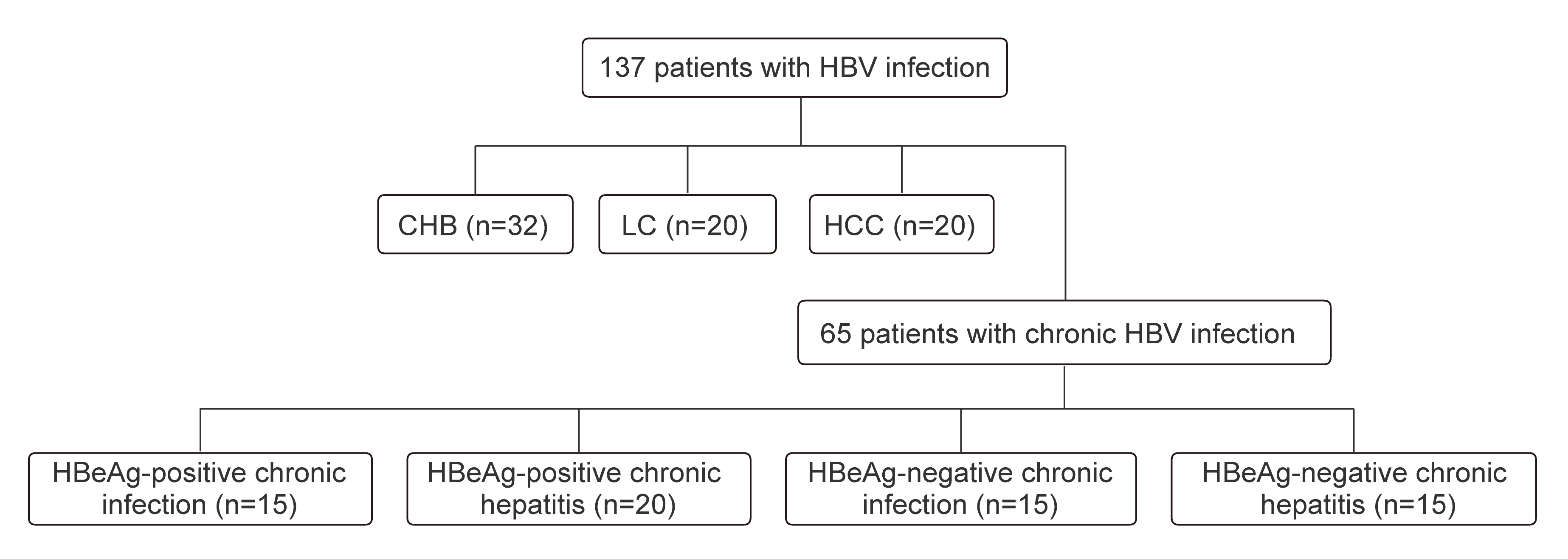


**Table S1. Baseline demographics and clinical characteristics of HBV infection-related diseases population**

| **Variables** | **Healthy**  **Control**  **（HC）** | **Chronic Hepatitis B**  **（CHB）** | **Cirrhosis of Liver**  **(LC)** | **Hepatocellular Carcinoma**  **(HCC)** |
| --- | --- | --- | --- | --- |
| **Number of participants** | 20 | 32 | 20 | 20 |
| **Clinical parameters** | | | |  |
| Gender, *n* Male/*n* Female | 13/7 | 23/9 | 15/5 | 16/4 |
| Age, years | 52±8 | 50±9 | 55±6 | 60±10 |
| **Laboratory parameters** | | | |  |
| Log_10_ HBV DNA, IU/ml | - | 6.5±1.7 | - | 5.4±0.9 |
| Log_10_ HBsAg, IU/ml | - | 3.9±0.9 | 2.3±1.6 | 2.0±1.6 |
| Log_10_ HBeAg, S/CO | - | 2.3±1.0 | -0.05±1.0 | -0.7±0.9 |
| ALT, U/L | 15±9 | 156±97 | 47±16 | 49±28 |

Data are mean ± SD unless otherwise indicated. HBV, hepatitis B virus; HBeAg, hepatitis B e antigen; HBsAg, hepatitis B surface antigen; ALT, alanine aminotransferase.

**Table S2. Baseline demographics and clinical characteristics of the natural history of chronic HBV infection population**

| **Variables** | **Healthy**  **Control**  **（HC）** | **HBeAg-positive**  **chronic HBV infection（Ⅰ）** | **HBeAg-positive**  **chronic hepatitis B（Ⅱ）** | **HBeAg-negative**  **chronic HBV infection**  **（Ⅲ）** | **HBeAg-negative**  **chronic hepatitis B**  **（Ⅳ）** |
| --- | --- | --- | --- | --- | --- |
| **Number of participants** | 20 | 15 | 20 | 15 | 15 |
| **Clinical parameters** | | | | | |
| Gender, *n* Male/*n* Female | 12/8 | 11/4 | 15/5 | 11/4 | 13/2 |
| Age, years | 50±9 | 39±6 | 47±9 | 47±6 | 50±8 |
| **Laboratory parameters** | | | | | |
| Log_10_ HBV DNA, IU/ml | - | 8.1±0.8 | 7.0±2.5 | - | 4.8±1.8 |
| Log_10_ HBsAg, IU/ml | - | 4.5±0.6 | 4.1±0.6 | 2.7±0.4 | 3.2±0.9 |
| Log_10_ HBeAg, S/CO | - | 3.6±0.7 | 2.6±1.2 | -0.5±0.1 | -0.4±0.2 |
| ALT, U/L | 19±7 | 26±8 | 192±97 | 25±12 | 99±26 |

Data are mean ± SD unless otherwise indicated. HBV, hepatitis B virus; HBeAg, hepatitis B e antigen; HBsAg, hepatitis B surface antigen; ALT, alanine aminotransferase.
